# Amebaborne “*Attilina* *massiliensis*” Keratitis, France

**DOI:** 10.3201/eid2402.170541

**Published:** 2018-02

**Authors:** Alexandre Battaini, Bernard La Scola, Gaëlle Ho Wang Yin, Louis Hoffart, Michel Drancourt

**Affiliations:** Aix Marseille Université, Marseille, France (A. Battaini, B. La Scola, M. Drancourt);; Aix-Marseille University–APHM, Hôpital de la Timone, Marseille (G.H.W. Yin, L. Hoffart)

**Keywords:** *Attilina* *massiliensis*, keratitis, ameba, amoeba, *Acanthamoeba* *castellani*, matrioshka strategy, corneal pathogen, *A*. *Castellani* Neff strain, *Serratia* *liquefaciens*, *Stenotrophomonas* *maltophilia*, bacteria

## Abstract

We report a case of *Acanthamoeba castellani* keratitis in a person who wore contact lenses. The amebae hosted an ameba-resistant bacterial symbiont, provisionally named “*Attilina massiliensis*,” a yet undescribed α-Proteobacterium.

Amebal keratitis is an aggressive ocular infection that can lead to blindness (*1*). It is usually associated with wearing soft contact lenses; Dart et al. documented that in countries with a high prevalence of contact lens wear, 85%–88% of *Acanthamoeba* keratitis cases occurred in contact lens users (*1*). These amebae host ameba-resistant bacteria, and increase their pathogenicity to the host (*2*). Ameba hosting intra-amebal microorganisms have been rarely documented in cases originating in contaminated contact lenses (*3*) and never in mixed keratitis. We report a case of mixed ameba–amebal-resistant bacterial keratitis.

A 17-year-old woman who wore contact lenses consulted the ophthalmology department of the clinic associated with Hôpital de la Timone, Marseille, France, in July 2016, after experiencing 1 month of keratoconjunctivitis symptoms related to an undocumented clinical diagnosis of herpes virus keratitis of the left eye. The patient had been prescribed a 1-week treatment with valacyclovir (3×/d) and a corneal dressing. Examination of the left eye showed 4/10 visual acuity; the right eye was normal. Slit-lamp examination showed a central radial keratoneuritis, central corneal edema, central diffuse infiltrate, and a punctate superficial keratitis with no predescemetic precipitates and no satellite lesions (Figure). The patient was admitted to the hospital and was administered hourly topical treatments of polyhexamethylene biguanide eye drops, hexamidine, and 1% atropine. The patient, whose diagnosis was early-stage *Acanthamoeba* keratitis infection, was discharged after 5 days of treatment; a corneal swab sample at discharge was negative for herpes virus, varicella zoster virus, adenovirus, enterovirus, cytomegalovirus, and *Chlamydia trachomatis*. Follow-up 7 days later yielded reduced symptoms. We followed up on the patient biweekly and slowly tapered drugs over 4 months; the previously negative pathogen tests remained negative. However, culture yielded *Corynebacterium ureicelerivorans*, identified on the basis of a 98.7% partial *rpo*B gene sequence similarity with the reference sequence (GenBank accession no FJ392018.1), *Acanthamoeba castellani*, identified on the basis of a 99% 18S rRNA gene sequence similarity with reference genotype T4 (GenBank accession no. U07416.1). Further culture of the amebal isolate in sterile peptone-yeast-extract-glucose broth (Culture-Top, Courtaboeuf, France) by using both optic microscopy and electron microscopy (Figure) yielded an intra-amebal *Holosporaceae* bacterium observed in the cytoplasm of the ameba. This symbiont, a yet undescribed α-Proteobacterium of the family of *Holosporaceae* that had been provisionally named “*Attilina massiliensis*,” was identified on the basis of a 100% 16S rRNA gene similarity with the reference sample (GenBank HM138368). After subculture, this “*A. massiliensis*” isolate was shown to be flagellated and highly mobile. Moreover, it was shown to lyse the *A. castellani* Neff strain (ATCC 30010), an observation suggesting motility-linked pathogenicity. Culturing the lens storage case yielded *Serratia liquefaciens* and *Stenotrophomonas maltophilia*, identified by matrix-assisted laser desorption/ionization time-of-flight mass spectrometry (*4*).

**Figure:**
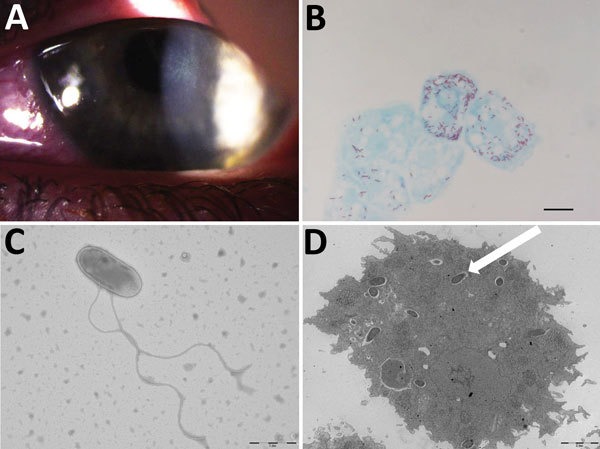
Results of testing for a 17-year-old woman with keratoconjunctivitis symptoms, Marseille, France, July 2016. A) Slit-lamp optic microscopic photograph of left eye infected with pseudo-dendritic keratitis associated with *Acanthamoeba castellani*–“*Attilina massiliensis*” ocular infection. B) Microscopic aspect of *A. castellani* ameba infected by “*A. massiliensis*” from corneal swab sample. Scale bar indicates 1 μm. C) Optic microscopy image of flagellated, free-living “*A. massiliensis*” from swab sample. Scale bar indicates 1 μm. D) Electron microscopy image of the ameba containing the “*A. massiliensis*” endosymbiont, stained by using Gimenez staining (white arrow). Scale bar indicates 2 μm.

In this patient, mixed *C. ureicelerivorans*, *A. castellani*, and “*A. massiliensis*” keratitis was firmly documented by the isolation and culture of the pathogens identified by using appropriate controls and validated protocols. The fact that we had never documented such an infection in our laboratory excludes mere false-positive results caused by contamination. *C. ureicelerivorans* has mainly been reported to cause septicemia, not ophthalmologic infections (*5**,**6*). Its sources and potential relationships with waterborne amebae are unknown. *Acanthamoeba* spp. amebae cause severe keratitis, which may cause visual loss (*1*). *Acanthamoeba* spp. amebae are ubiquitous in tap water (*7*). Tap water could be a source of contamination of contact lenses through the wearer’s handwashing habit before lens manipulation (*8*). Wide varieties of amebae have been documented in contaminated contact lenses, eventually leading to amebal keratitis outbreaks (*9*). 

Culturing an *A. castellani* ameba isolated from a diseased cornea yielded “*A. massiliensis*,” which we isolated once 8 years ago from an *Acanthamoeba polyphaga* ameba collected from a contact lens storage case that belonged to a patient unrelated to the case-patient we report here. The potential for this emerging ameba-resistant bacterium to cause keratitis remains to be analyzed, but we observed that this mobile symbiont lysed the reference amebal strain, demonstrating its cytopathogenicity. Also, ameba-resistant organisms do comprise acknowledged opportunistic pathogens (*2*), and corneal toxicity was previously reported as significantly higher for *Acanthamoeba*-hosting endosymbionts (*10*). This investigation illustrates that amebae present in cases of keratitis may shelter organisms that should be provisionally regarded as potential opportunistic pathogens under these circumstances.
